# Improving Medication Safety for Patients with Adrenal Insufficiency Using Electronic Health Record Optimization

**DOI:** 10.1097/pq9.0000000000000939

**Published:** 2026-10-28

**Authors:** Meenal Gupta, Alyssa Huang, Kate Ness, Kristen Carlin, Lori Rutman

**Affiliations:** From the * Department of Pediatrics, Division of Endocrinology, University of Washington School of Medicine, Seattle, Wash.; † Center for Clinical and Translational Research, Seattle Children’s Research Institute, Seattle, Wash.; ‡ Epidemiology and Analytics for Research Core, Seattle Children’s Research Institute; § Department of Pediatrics, Division of Emergency Medicine, University of Washington School of Medicine, Seattle, Wash.

## Abstract

**Introduction:**

Life-threatening adrenal crisis is preventable with timely administration of a stress dose of hydrocortisone, but recognition is often delayed. Sentinel patient safety events in 2020 highlighted the need to standardize adrenal insufficiency management. The primary aim of our quality improvement project was to reduce adrenal insufficiency medication errors in the emergency department (ED) by 10% from the baseline in 2 years.

**Methods:**

We included patients younger than 21 years with adrenal insufficiency and a prescription for parenteral hydrocortisone sodium succinate or oral hydrocortisone (prescribed by endocrinology) seen at our hospital between January 1, 2019, and September 30, 2023. Electronic health record (EHR) optimization changes included usage of a best practice advisory to improve visibility of adrenal insufficiency diagnosis and standardization of orders, steroid action plans, and education documentation. ED medication errors were defined as ED encounters with an emergency service index score of 1 or 2 with suboptimal stress dose (<50 mg/m^2^ of hydrocortisone sodium succinate) administration. The secondary outcome measure was safety reports related to medication errors in the ED or ambulatory setting. We analyzed outcome measures using statistical process control and tracked usage rates of EHR changes.

**Results:**

We included 776 patients and 920 ED encounters. ED medication errors reduced from a baseline of 21% to 13.9% by study end. The interval between safety reports increased from an average of 74.4 to 775 days. Process measure use was 60% or greater for the problem list, best practice advisory, and steroid action plan, and 34% for education documentation.

**Conclusions:**

We successfully leveraged EHR optimization to standardize adrenal insufficiency management, increase visibility of critical information, and significantly reduce medication errors.

## INTRODUCTION

Adrenal crisis is a life-threatening condition that can be prevented through timely recognition and management using stress-dose steroids. Despite the availability of effective treatment, the annual estimated incidence of adrenal crisis is 5–10 episodes per 100 patient-years, with 1 in 200 episodes resulting in death. Nonspecific symptoms leading to a variable presentation and clinician and caregiver knowledge gaps have been identified as contributing factors to missed diagnoses and delayed treatment.^1–5^

In 2020, 2 sentinel cases at our institute involving delayed diagnosis and treatment of adrenal insufficiency revealed gaps in caregiver education as well as prescription errors. Case 1 was a 4-year-old patient with central adrenal insufficiency who had delayed administration of stress-dose steroids during illness due to the caregiver’s under-recognition of illness severity and need for urgent treatment. Case 2 was a neonate with congenital adrenal hyperplasia who was prescribed one-third of the appropriate dose of oral hydrocortisone without written stress-dose instructions in the prescription. Although stress-dose education and an action plan were provided to the family, this was not documented in the patient’s medical chart. At 1 month of age, routine laboratory evaluation revealed electrolyte derangements consistent with adrenal crisis, warranting hospitalization for treatment. Similar cases have been reported across institutions, underscoring a recurring systems-level challenge in the care of patients with adrenal insufficiency.^1,3,6^

The gaps here are 2-fold: early recognition and effective management. Published guidelines describe a multifaceted approach to address these gaps, including active and continuous patient and family education, clinician education, and electronic health record (EHR)–based tools, such as adrenal insufficiency alerts, order sets, templated patient instructions, and easily accessible stress-dose plans.^1–3^ The effectiveness of computerized order entry and clinical decision support (CDS) systems in reducing medication prescription errors is well established.^7,8^ CDS, like a best practice advisory (BPA), has been used to increase visibility of life-threatening conditions such as sepsis and acute kidney injury. Although these studies have improved process measures, they have not consistently improved clinical outcomes.^9–11^ For pediatric adrenal insufficiency, studies have been limited to evaluating either outcome or process measures in a single setting. Halpin et al^6^ demonstrated improved stress-dose management of adrenal crisis in the emergency department (ED) through use of CDS. In the endocrine clinic setting, automated alerts and “smart buttons” linked to templated instructions improved documentation of stress-dose teaching and plan, respectively.^12,13^ However, studies on the overall effectiveness of EHR-based tools across care teams managing patients with adrenal insufficiency are lacking.

This quality improvement (QI) project is the first study describing the effectiveness of a multipronged approach using comprehensive EHR-based optimization to improve early recognition and management of adrenal insufficiency. Our primary aim was to reduce medication errors related to adrenal insufficiency in the ED setting by 10% from the baseline within 2 years. Our secondary aim was to reduce the self-reported adrenal insufficiency medication errors in the ED and ambulatory setting.

## METHODS

A multidisciplinary QI team was assembled, including 3 pediatric endocrinologists (including the project lead), a clinic operations manager, an ED physician, and information technology (IT) analysts. The team developed a driver diagram to identify key areas for improvement (Fig. 1).

**Fig. 1.**
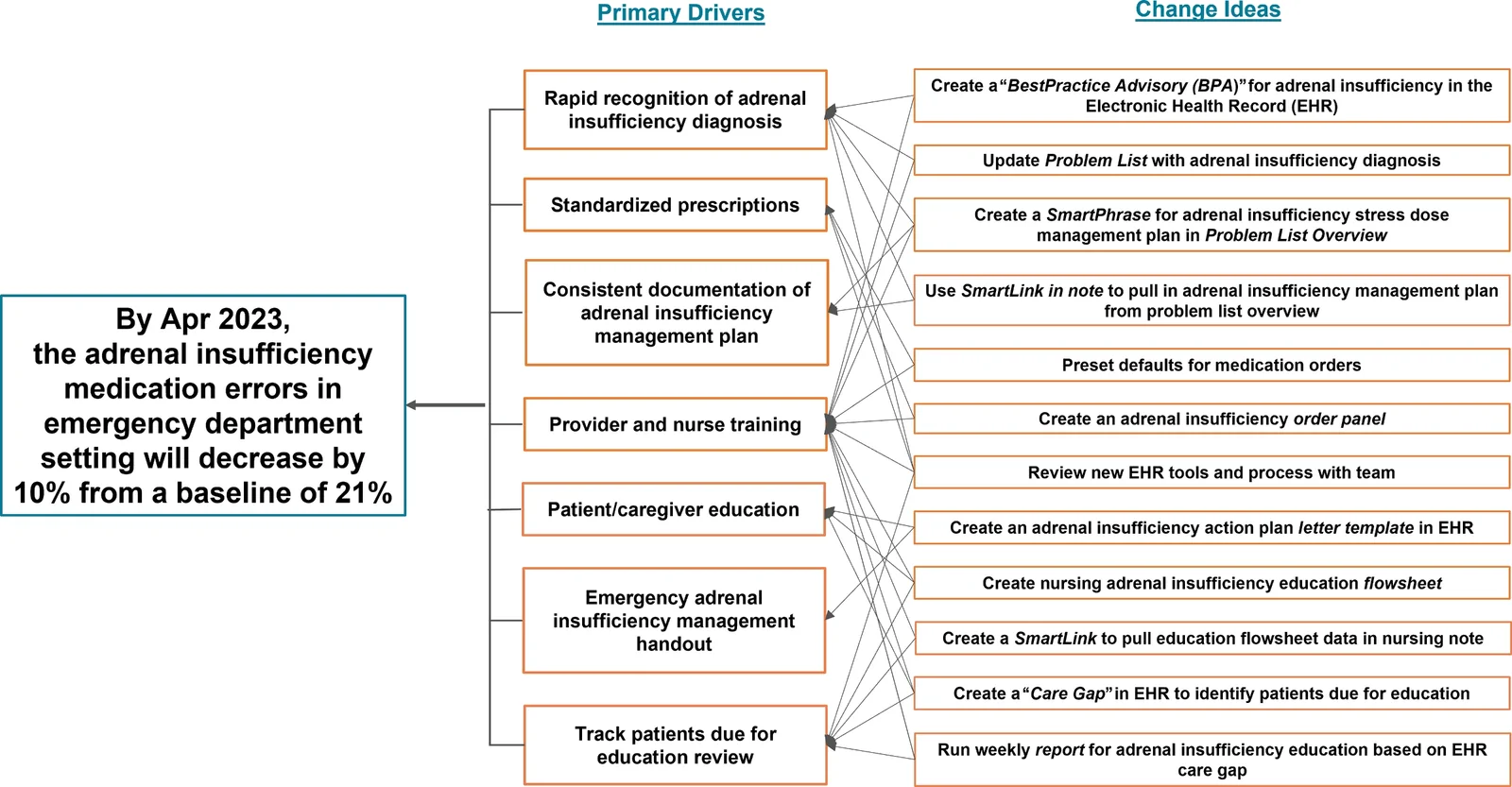
Driver diagram

### Context

This study was conducted in a tertiary care, freestanding children’s hospital with a 5-state referral base for pediatric endocrinology care. The pediatric endocrinology group consists of 22 physicians and 2 advanced practice providers who care for approximately 400–500 patients diagnosed with adrenal insufficiency per year. Patients with adrenal insufficiency are seen in the ED, inpatient, and ambulatory settings. Endocrinology follows all patients with primary and permanent central adrenal insufficiency and those with known or suspected glucocorticoid-induced adrenal insufficiency (GIAI). Some subspecialties, such as the bone marrow transplant team, manage GIAI independently in their cohort of patients with graft-versus-host disease. The institute’s EHR switched from Cerner to Epic on October 3, 2020. The hospital uses an electronic patient safety and experience self-reporting system called “eFeedback,” whereby any healthcare team member can report concerns or incidents related to patients’ safety. These eFeedbacks are routed to appropriate leaders for follow-up, investigation, and documentation of a resolution summary. Specific data fields and filters allow analysis of medication-safety errors. During the baseline period, this patient safety self-reporting system was active. However, the EHR had no flag or alert to identify patients with adrenal insufficiency, and the management plan was documented only in the clinic note. No specific adrenal insufficiency order panel existed. The adrenal insufficiency action plan template on the institute’s intranet was customized to create individual care plans for families, but these were not scanned into the EHR and were therefore not available for review by all care teams. Moreover, the nursing team did not use a standardized method of documentation of patient/caregiver education.

### Study Population

Patients aged 21 years or younger with International Classification of Diseases, Tenth Revision and SNOMED-CT (Systematized Nomenclature of Medicine Clinical Terms) diagnostic codes for primary and secondary adrenal insufficiency who had (1) a current or past prescription of intravenous (IV) or intramuscular (IM) hydrocortisone sodium succinate (HSS) prescribed by any clinician or (2) oral hydrocortisone prescribed during an endocrine clinic visit only were included. Parenteral HSS is typically only prescribed for treatment of adrenal insufficiency and could be prescribed by any clinician (eg, at discharge from the ED). However, because oral hydrocortisone is prescribed for a variety of diagnoses besides adrenal insufficiency, only prescriptions linked to an endocrine clinic visit were included. An endocrinology clinic visit was not required.

### Study Timeline

The baseline study period was January 1, 2019, to September 30, 2020, and the intervention period was October 1, 2020, to September 30, 2023.

### Interventions

The following EHR-based interventions were implemented:

1. Creation of an EHR BPA linked to problem list entry of adrenal insufficiency (October 2020) (Fig. 2A).
2. Endocrine providers were instructed to enter the appropriate diagnosis in the problem list, which would automatically fire the passive BPA on the patient’s storyboard. This was visible across all patient settings (ambulatory, inpatient, and ED) during an active encounter and was meant to immediately alert all healthcare team members of the patient’s risk of adrenal suppression.
3. An EHR SmartPhrase to document stress-dose management plan in the problem list overview (October 2020) (Fig. 2B).
4. Endocrine providers used a SmartPhrase, which is a structured text expansion tool within Epic, to document the patient’s physiological and stress-dose plan within the “problem list overview,” which would allow easy access to the plan by hovering over the problem list using a computer mouse.
5. Adrenal insufficiency order panel, which included a combination of laboratory, medication, and supply orders with preset defaults (December 2020) (Fig. 3A).
6. A customized ambulatory order panel made commonly ordered laboratory tests, medications, and supplies for patients with adrenal insufficiency easy to access. Preset default settings for oral hydrocortisone included 3-times-a-day dosing and required free-text instructions for stress dosing (Fig. 3B). For emergency home management, the HSS prescription had a preset IM route of administration and supplies necessary for reconstitution and injection. We also created an inpatient version of this panel.
7. An adrenal insufficiency action plan for stress-dose management available as a letter template in the EHR (December 2020) (Fig. 4).
8. A patient-friendly action plan outlining patients’ physiological or daily dosing in green, moderate stress dosing in yellow, and major stress dosing in red, along with indications for when to use each dose, was created (Fig. 4A). This was accessible during a clinic encounter as a “speed button” letter template with autopopulated patient demographic information (Fig. 4B). The second section of the action plan included an emergency response card that could be provided to an outside ED or emergency medical services personnel (Fig. 4C). The initial letter template format posed challenges because it required printing in landscape orientation. Following feedback from the nursing team, the design was updated to a portrait-oriented, printer-friendly version in September 2021.
9. Nursing education flowsheet for adrenal insufficiency (March 2022). (**See figure, Supplemental Digital Content 1**, which displays the adrenal insufficiency nursing education flowsheet, https://links.lww.com/PQ9/A833.)
10. To enable discrete documentation of the topics covered and the date of patient/caregiver education conducted by the endocrine nursing team, we built a custom nursing education flowsheet. This information could be pulled automatically into a nursing note via a dynamic data field called “SmartLink”

**Fig. 2.**
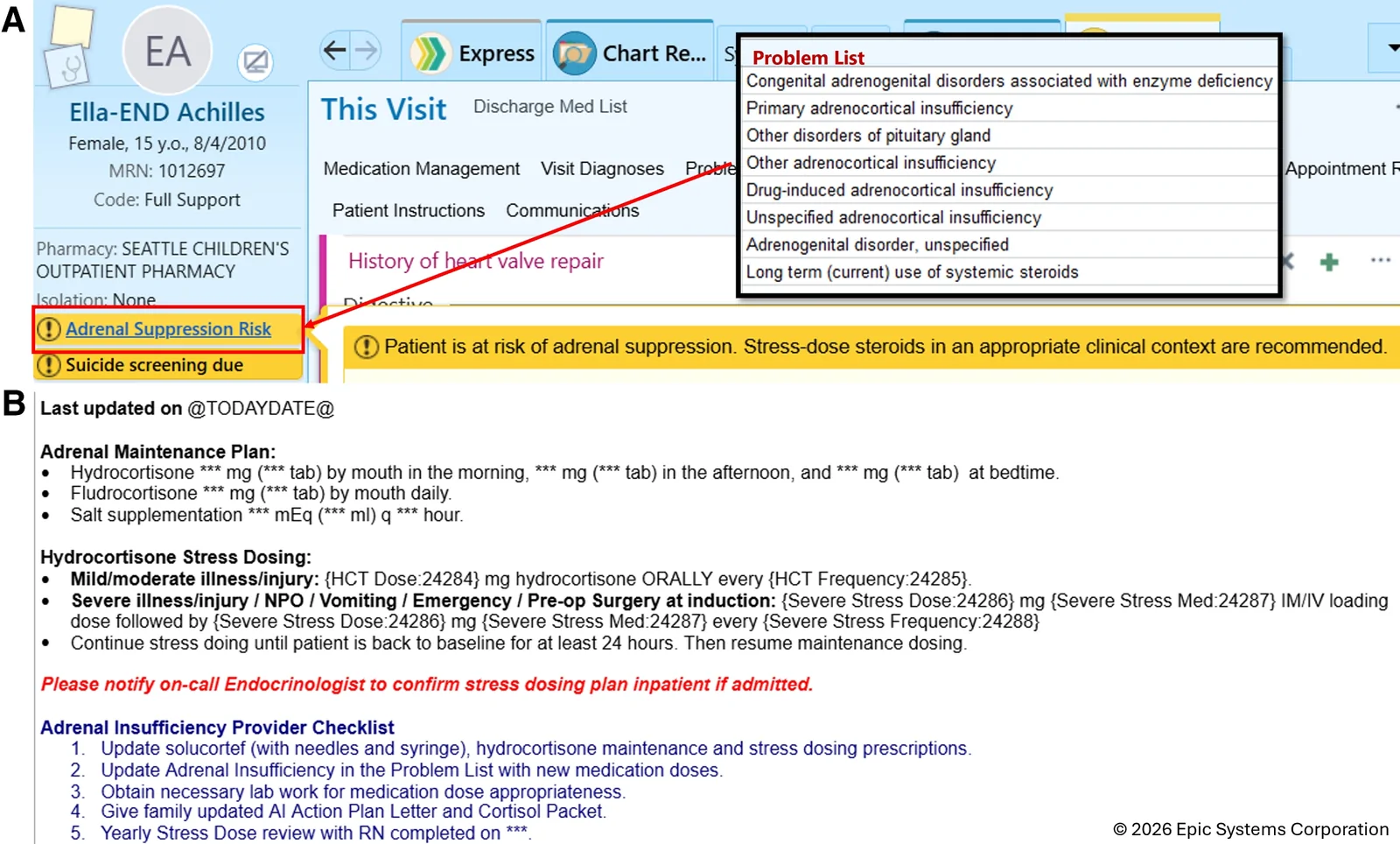
Adrenal insufficiency BPA and stress-dose management plan linked to problem list. A, BPA linked to a problem list entry. B, EHR SmartPhrase to document the stress-dose management plan in problem list overview

**Fig. 3.**
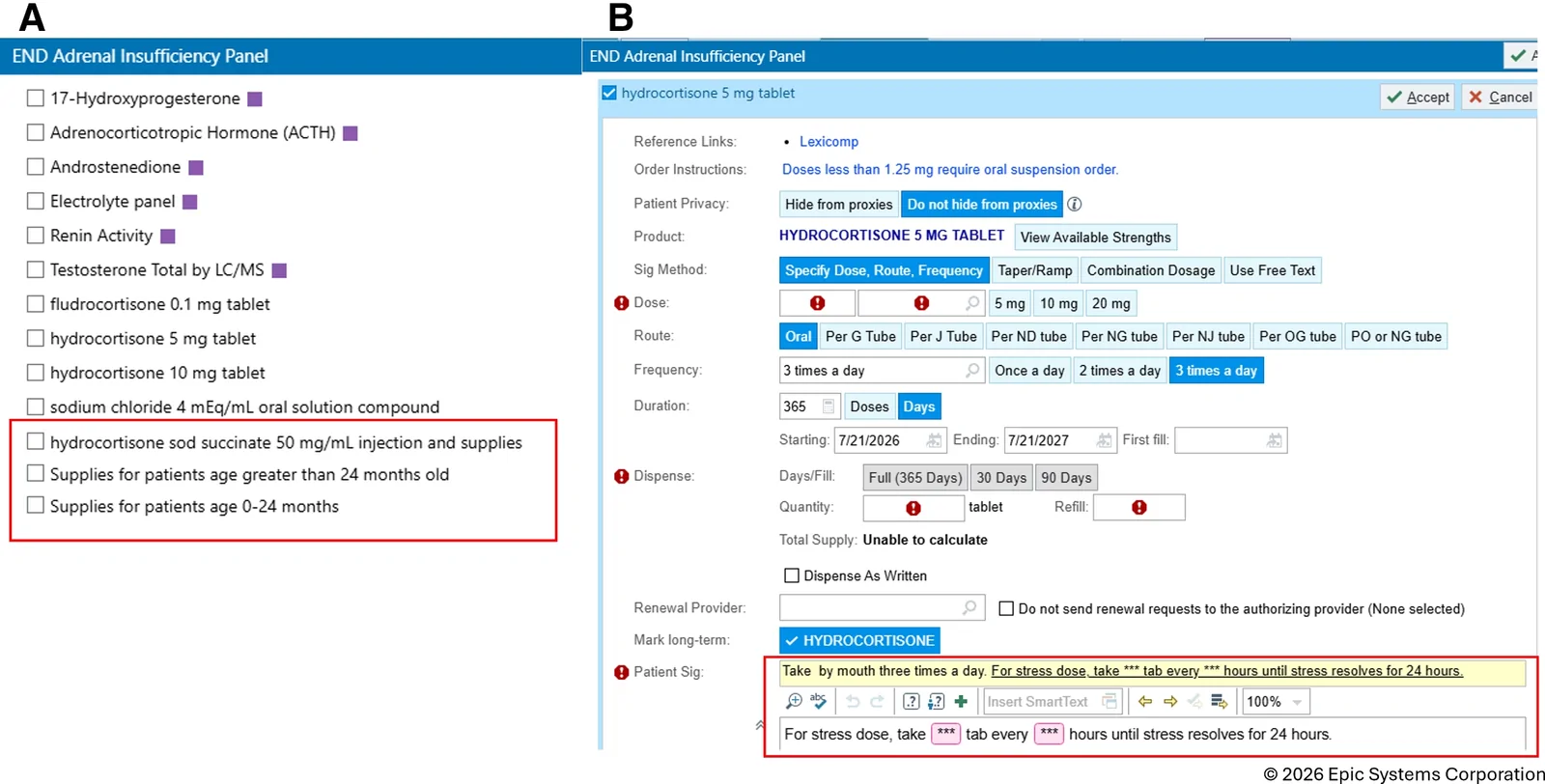
Adrenal insufficiency order panel including preset defaults and free text for stress dose management plan. A, Adrenal insufficiency order panel. B, Hydrocortisone order within the adrenal insufficiency order panel with free-text instructions for stress dosing.

**Fig. 4.**
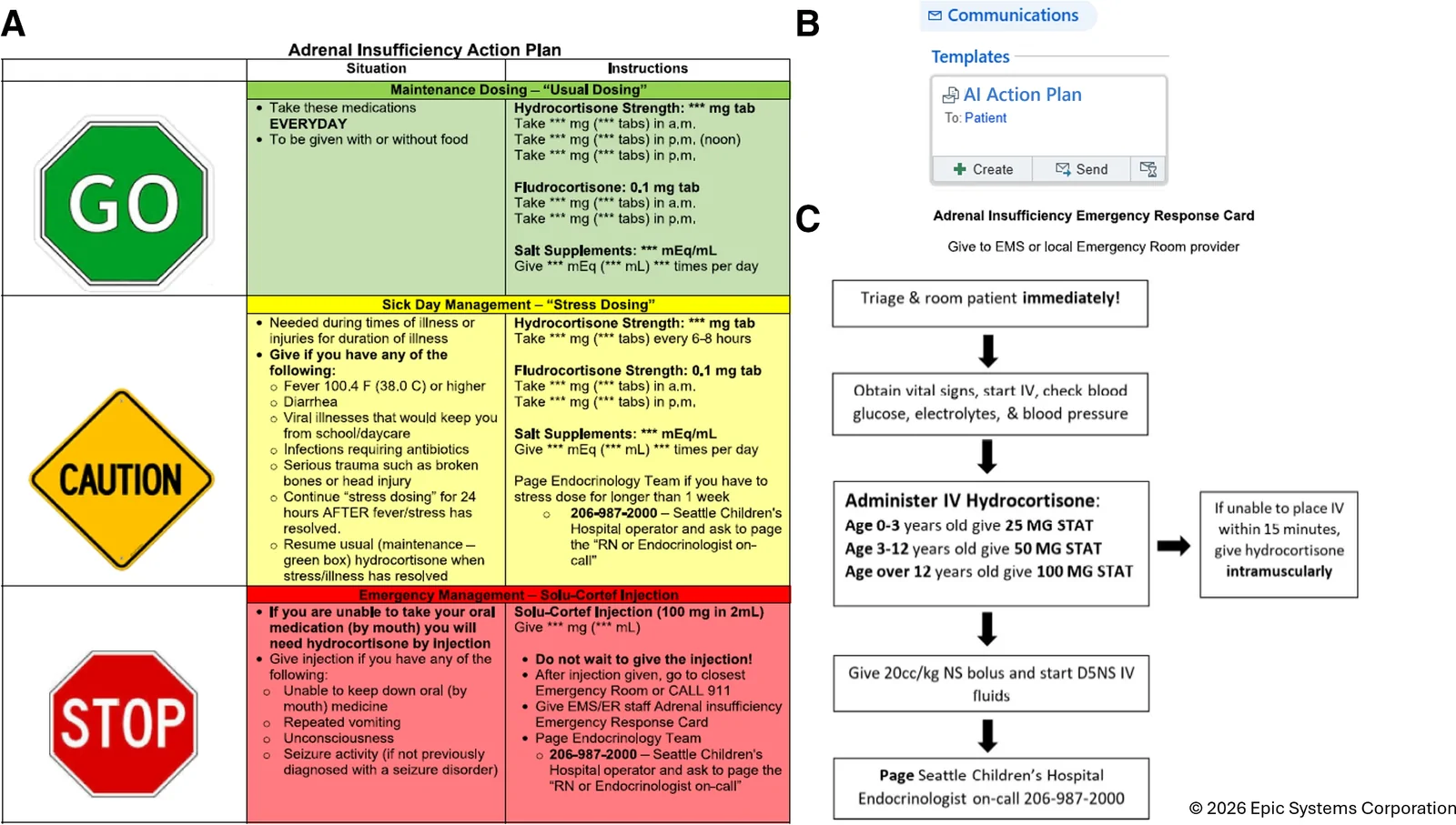
Adrenal insufficiency action plan letter and EHR tools. A, Adrenal insufficiency action plan. B, Speed button for the adrenal insufficiency action plan letter template. C, Adrenal insufficiency emergency response card. EMS, Emergency Medical Services; IV, intravenous; mg, milligrams; NS, normal saline.

### Educational Sessions with Faculty and Staff

The endocrinology team participated in several training sessions to prepare for the transition to the new EHR. In addition, specific educational sessions reviewed workflows related to the newly created EHR tools, supplemented by job aids outlining the specific steps. Although no directed education was provided to nonendocrinology providers, the ED team was trained to review critical information on the patient’s storyboard, and all providers were encouraged to keep the problem list updated (intervention 1) as part of the new EHR onboarding. All providers also had access to the new order panel.

### Measures

The primary outcome measure was the rate of medication errors related to adrenal insufficiency in the ED, measured over time. The numerator was the number of encounters of critically ill ED patients with an emergency service index (ESI) score of 1 or 2 in which a suboptimal hydrocortisone stress dose was administered. The denominator was all ED encounters of study patients triaged as an ESI level 1 or 2; ESI levels are available as discrete data in the EHR. Appropriate stress dosing was defined as either an HSS dose of 50 mg/m^2^ or more (body surface area-based dosing) or age-based dosing of 25 mg or more, 50 mg or more, and 100 mg or more for age ranges 0–5, 6–12 and 13 years or older, respectively.^5^ For patients with a BSA of 1.7 m^2^ or less, the administered dose in milligrams was divided by BSA to determine whether it exceeded 50 mg/m^2^. For patients with a BSA greater than 1.7 m^2^, the administered dose was used without adjustment. Encounters where the appropriate dose was not administered contributed to the medication error rate. The secondary outcome measure was the number of eFeedbacks related to adrenal insufficiency medication errors in the ED and ambulatory setting. We manually reviewed all eFeedback reports related to hydrocortisone use during the study period and narrowed them to those specific to adrenal insufficiency. We counted reports involving dose omission, prescription errors, or gaps in education in the ED or ambulatory setting as medication errors. We excluded medication errors in the operating room and inpatient units.

Because we primarily used process measures during endocrinology clinic visits, the denominator included patients seen in the endocrine clinic who had an IM/IV HSS or oral hydrocortisone prescription in the year before the clinical encounter. Three process measures were reviewed on a quarterly basis, and the numerator for each was derived using the percentage of study patients who had the respective EHR tool documented: (1) adrenal insufficiency problem list diagnosis, (2) adrenal insufficiency BPA, and (3) adrenal insufficiency action plan. The goal for adrenal insufficiency patient/caregiver education was to be completed annually, and we tracked this using the adrenal insufficiency nursing education flowsheet in the charts. For a balancing measure, we reviewed changes in the median ED length of stay (LOS) from baseline to the intervention period.

### Analysis

We summarized encounter-level, self-reported sociodemographic data and clinical features using means, SDs, medians, and interquartile ranges for continuous variables. We summarized categorical variables using frequencies and percentages. We analyzed ED medication errors bimonthly using a statistical process control p-chart to identify special-cause variation. Because eFeedback errors were rare, the number of days between consecutive errors was plotted on a t-chart. The final interval remained open at the end of the study period and was excluded from the control limit calculations. We applied control chart rules as described by Provost and Murrary.^14^ We recalculated the centerline when any of these rules indicated a nonrandom signal, including a run of 8 or more consecutive points above or below the baseline centerline. We described process measures with counts and percentages and analyzed changes in ED LOS using the Wilcoxon rank-sum test.

### Ethical Considerations

The institutional review board at our institute reviewed and approved this study through expedited review.

## RESULTS

We included 776 patients and 920 ED encounters in the analysis (Table 1). Most participants were younger than 13 years (72%), non-Hispanic White (47%), and had English as their preferred language of care (89%), with similar distributions by sex (42% female) and insurance (public 49%). The rate of ED medication errors decreased from 21% at baseline to 13.9% by the end of the study period, with a special-cause variation noted in November 2020 (Fig. 5). We reviewed 122 eFeedbacks, of which 37 related to adrenal insufficiency. After excluding the inpatient setting (n = 25) and operating room (n = 3), there were 6 eFeedbacks in the baseline period and 3 in the intervention period for the ED and ambulatory setting. The intervals between consecutive errors lengthened over time, reaching 775 days at study end, exceeding the average interval of 74.4 days, and no special-cause variation was detected (Fig. 6).

**Table 1.** Patient Demographics and Encounter Characteristics

Characteristic	Category	n (%)
Age, y	0–5	179 (23.1)
	6–12	232 (29.9)
	13–17	187 (24.1)
	18+	178 (22.9)
Sex	Female	336 (43.3)
	Male	440 (56.7)
Race	Asian	72 (9.3)
	Black	26 (3.4)
	Hispanic	159 (20.5)
	Multiracial/ethnic	51 (6.6)
	Native Hawaiian/Pacific Islander/American Indian/Alaska Native	6 (0.8)
	Non-Hispanic White	379 (48.8)
	Unknown/refused/not listed	83 (10.7)
Language	English	692 (89.2)
	Language other than English	84 (10.8)
Insurance	Other	23 (3)
	Private	408 (52.6)
	Public	345 (44.5)
Encounter type	ED encounter	311 (40.1)
	Endocrine encounter	465 (59.9)
ESI level, ED encounters	1	25 (8)
	2	286 (92)

**Fig. 5.**
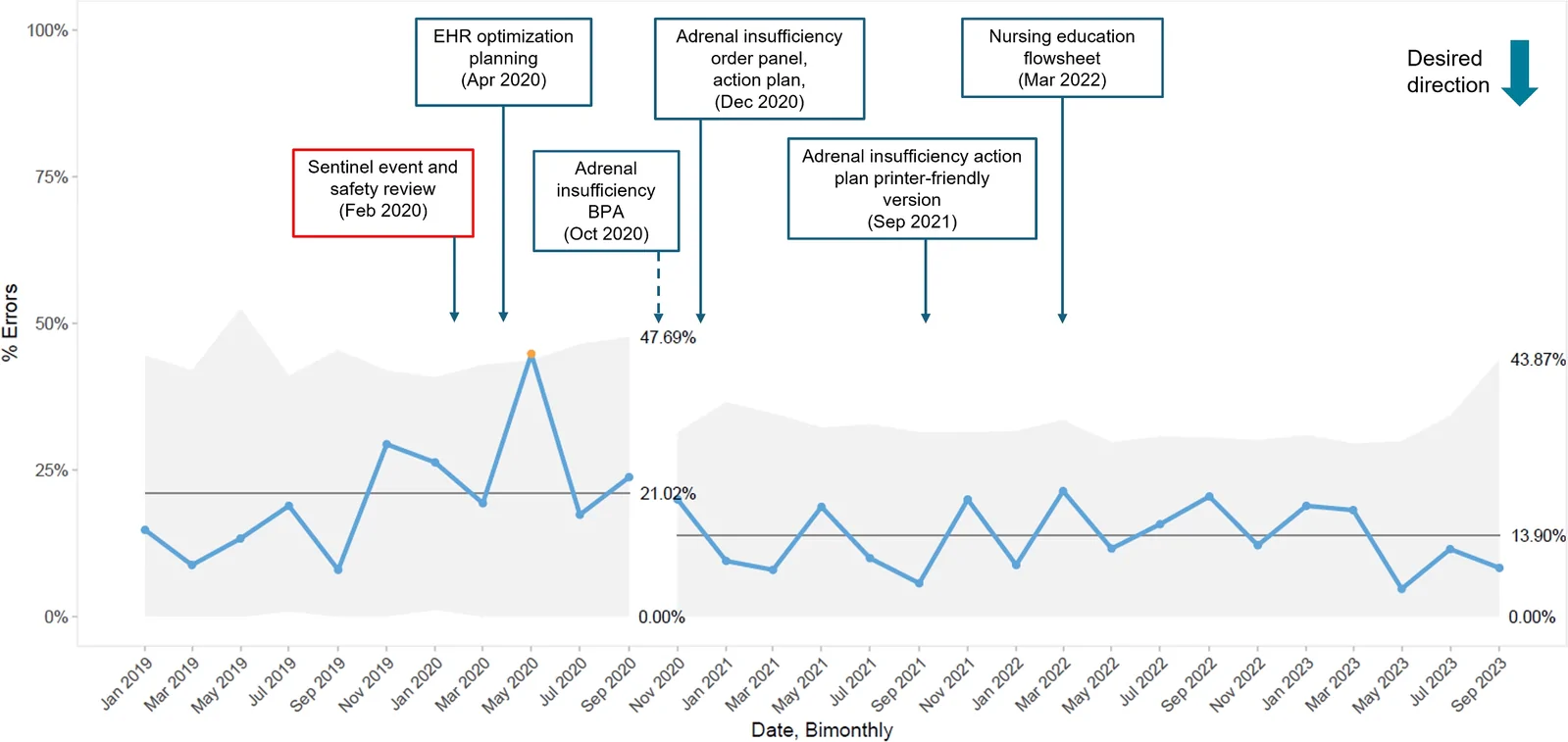
P-chart showing adrenal insufficiency ED medication errors over time. Special-cause variation was noted in November 2020, with 8 consecutive points falling below the baseline centerline of 21%, causing a centerline shift.

**Fig. 6.**
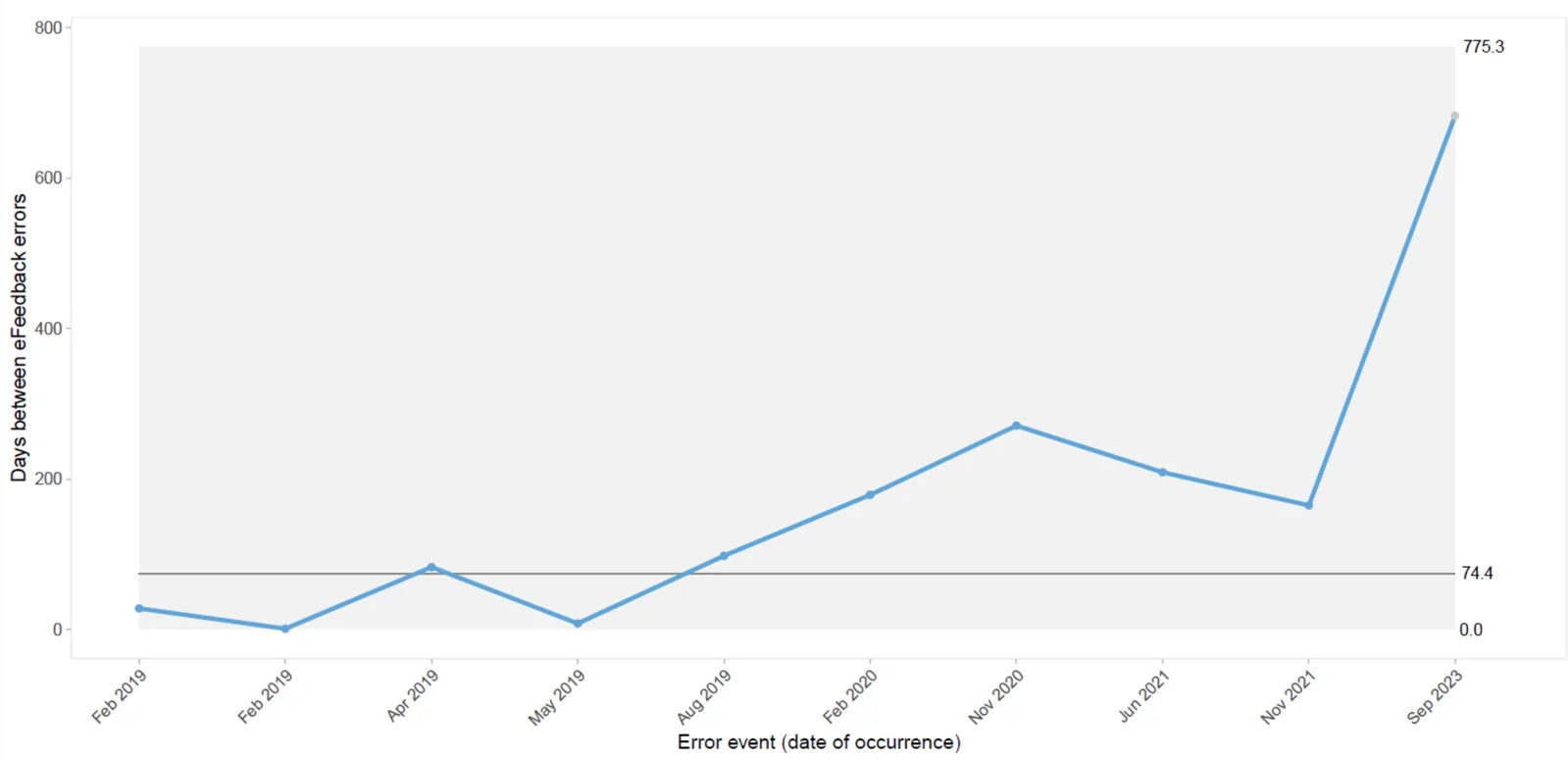
T-chart showing the eFeedback-free interval over time for adrenal insufficiency eFeedback errors in the ED and ambulatory settings.

The percentage of patients with adrenal insufficiency BPA documented increased from a baseline of 0% to 75%. Problem list and action plan documentation increased from 9% to 62% and from 0% to 66%, respectively. The percentage of patients with annual education documented in the nursing flowsheet increased from a baseline of 0% to 34%. The median ED LOS increased from baseline 239.5 (150.5) to 271 (172) minutes in the intervention period (*P* = 0.02).

## DISCUSSION

Using EHR optimization implemented across all patient care settings, we reduced the medication error rate by 34%, exceeding our primary aim of a 10% reduction. Timely recognition of adrenal suppression risk using the BPA in a highly visible section of the patient’s chart was the primary contributor to this improvement, as shown by the timing of the special-cause variation soon after BPA implementation. In addition, embedding the emergency management plan in the problem list overview and standardizing prescription orders and patient/caregiver education likely helped sustain this improvement.

Despite its single-center design, this study includes the largest patient cohort to date analyzing medication safety in adrenal insufficiency. Halpin et al^6^ described using a similar passive CDS tool called a “critical information note” to outline a generic emergency management plan for adrenal insufficiency across 145 encounters (79 patients). They report a higher likelihood (53%) of appropriate hydrocortisone dose administration in the ED for adrenal crisis than at baseline (20%; *P* = 0.02) after implementation.^6^ However, several key differences exist between their study and ours. Our study population is 10 times larger than theirs. We developed individualized stress-dose management plans and documented them in 2 places: the problem list overview (Fig. 2B) and a patient-friendly action plan (Fig. 4). Their passive CDS tool documentation rate was much lower (46%) than our BPA use (75%) and action plan use (66%). We also used other EHR optimization tools, such as standardized order panels and nursing documentation, to improve overall management. And although numbers were low, we reviewed eFeedback data as an additional outcome measure to capture errors in the ambulatory and ED settings.

Chakravarthy et al^12^ used QI to standardize documentation of individualized stress-dose plans using SmartPhrases and “smart buttons” but achieved only a 45% utilization rate. Their stress-dose plan was documented in endocrine clinic notes, unlike our use of the problem list overview and stress-dose action plan letter, which are more accessible locations for nonendocrinologists. Moreover, although their goal of improved documentation was to extend this information to nonendocrine providers, the impact on stress-dose administration in the ED or other clinical outcomes was not studied.

Schweiger et al^13^ used active CDS in the endocrine clinic to improve documentation of stress-dose education from 38% to 68% in a cohort of 128 patients. However, their alert acknowledgment rate was only 45%. They also describe the use of similar alerts in the ED setting, but the effectiveness of either alert in improving patient outcomes was not reported. In contrast, our endocrine nurses create a patient-facing action plan for stress-dose education and use an education flowsheet to document completed education. Education flowsheet use was lower (34%), whereas action plan use was higher (66%). Our nursing team likely perceived this additional flowsheet documentation as burdensome. Another key difference is that the active alert in their study was linked to oral or IV steroid prescriptions, which resulted in a 13% erroneous firing rate.^13^ Because steroids are prescribed by several specialties for various diagnoses, accurate identification of patients who have adrenal insufficiency remains challenging. As a result, our design required a clinician-created adrenal insufficiency problem list entry. This added an extra step to bring up the BPA but improved accuracy.

Several studies have shown an increase in medication errors and safety reports following EHR transition.^15–17^ In contrast, our most successful intervention for error reduction was implementing the BPA, and it was closely tied to EHR transition time. A proactive approach following a sentinel patient event to identify areas for improvement, along with targeted educational sessions, live demonstrations of CDS tools, and real-time EHR superuser support improved buy-in from clinicians and was instrumental to our success. We achieved a 60% or greater use for 3 of the 4 EHR changes (interventions 1, 3, and 4), compared with 45% in the aforementioned studies.^6,12,13^ Special attention to CDS design also played an important role in translating fidelity with process measures into meaningful clinical improvement. CDS tools can be active, interrupting clinician workflow, or passive, visible but not interruptive. Use of active BPAs has been associated with much higher odds of recommended action being taken,^18^ but concerns about alert fatigue remain, and replacement with passive CDS is recommended when appropriate.^19^ Studies on the effectiveness and usage of passive CDS have shown variable results.^20,21^ However, our passive BPA design was intentional and based on clinician workflows and user-centered principles outlined in the “5 rights” framework.^22^ Using the problem list allowed a one-time chart update to automatically create the passive BPA, with visibility across all encounters. Moreover, displaying the BPA on the storyboard aligned with the ED team’s workflow for accessing critical patient information. The median ED LOS increased by 31 minutes in the intervention period. Although we did not systematically analyze the many variables affecting LOS, this may reflect additional time needed to administer the appropriate stress dose.

In addition to the strengths noted earlier, the EHR optimization tools used in our patient cohort are broadly generalizable to patient conditions that require high visibility of critical information, standardized orders, and education. These are also standard CDS tools available across different EHR vendors and are not necessarily limited to Epic. Generalizability, however, may be limited by IT infrastructure and institutional resources related to implementation, such as education, design modification through iterative feedback, and at-the-elbow support. Attention should also be paid to institutional practices around active versus passive CDS, and objective analyses of utilization rates and clinical outcomes are strongly recommended.

Our study has some limitations. Given the large study population, we did not review individual patient charts. Thus, there is potential for over-inclusion of patients in the medication error group even though they may not qualify for a stress dose. Examples include patients whose GIAI had resolved by the time of the ED visit and those who received IM HSS at home or en route to the ED. Despite being the most common form of adrenal insufficiency, the risk of GIAI remains largely under-recognized, with more than 70% of cases identified during acute hospitalizations.^23^ GIAI may resolve and recur based on high-dose steroid use; thus, we erred toward being overly inclusive so as not to miss any GIAI-related medication errors. Anecdotal reports indicated consistent use of the adrenal insufficiency order panel at initial endocrine clinic visits, but refill prescriptions sent using existing orders precluded objective analysis of order-panel use during the study period. Efeedbacks, as our secondary outcome measure, were meant to capture medication errors in the ambulatory setting in addition to the ED, but their overall numbers were small. Although our pharmacy has a robust system of documenting any medication-related errors as eFeedbacks, errors related to inaccurate prescriptions to outside pharmacies were not captured unless they resulted in an ED visit or an eFeedback.

In conclusion, comprehensive EHR optimization aimed at addressing all gaps in care related to adrenal insufficiency, coupled with thoughtful implementation, resulted in higher-than-average utilization rates for all EHR tools and significantly improved medication safety for patients with adrenal insufficiency. As a next step, we created an automated EHR report based on nursing education documentation to identify patients due for annual education. Periodic use of this report has supported clinic staffing capacity planning, ensuring nurses are available to address education gaps during patients’ existing clinic appointments. Efforts to improve flowsheet use are ongoing. We are also scaling and spreading this QI initiative to improve management of GIAI through collaboration with specialties prescribing high-dose steroids, such as rheumatology and gastroenterology. As noted previously, GIAI remains under-recognized, and recent surveys of neonatologists and rheumatologists have further demonstrated important knowledge gaps and infrequent investigation for GIAI.^23–25^ Given limited access to pediatric endocrinologists, providing resources to and partnering with these specialties is crucial to improving GIAI management.^26,27^ We hope other institutions can use these tools to improve care and streamline management for all patients with adrenal insufficiency.

## ACKNOWLEDGMENTS

The authors acknowledge the institutional IT team, in particular, analyst Susan Yu, for creating all EHR tools, as well as the clinical team for support and adoption of these tools.

## Supplementary Material
