# Supplementary material for: Improving Medication Safety for Patients with Adrenal Insufficiency Using Electronic Health Record Optimization
Source: Pediatr Qual Saf. 2026 Oct 28;11(6):e939. doi: 10.1097/pq9.0000000000000939 (PMC13716407; doi:10.1097/pq9.0000000000000939)
Supplement: Supplementary file 1 [file pqs-11-e939-s001.pdf]

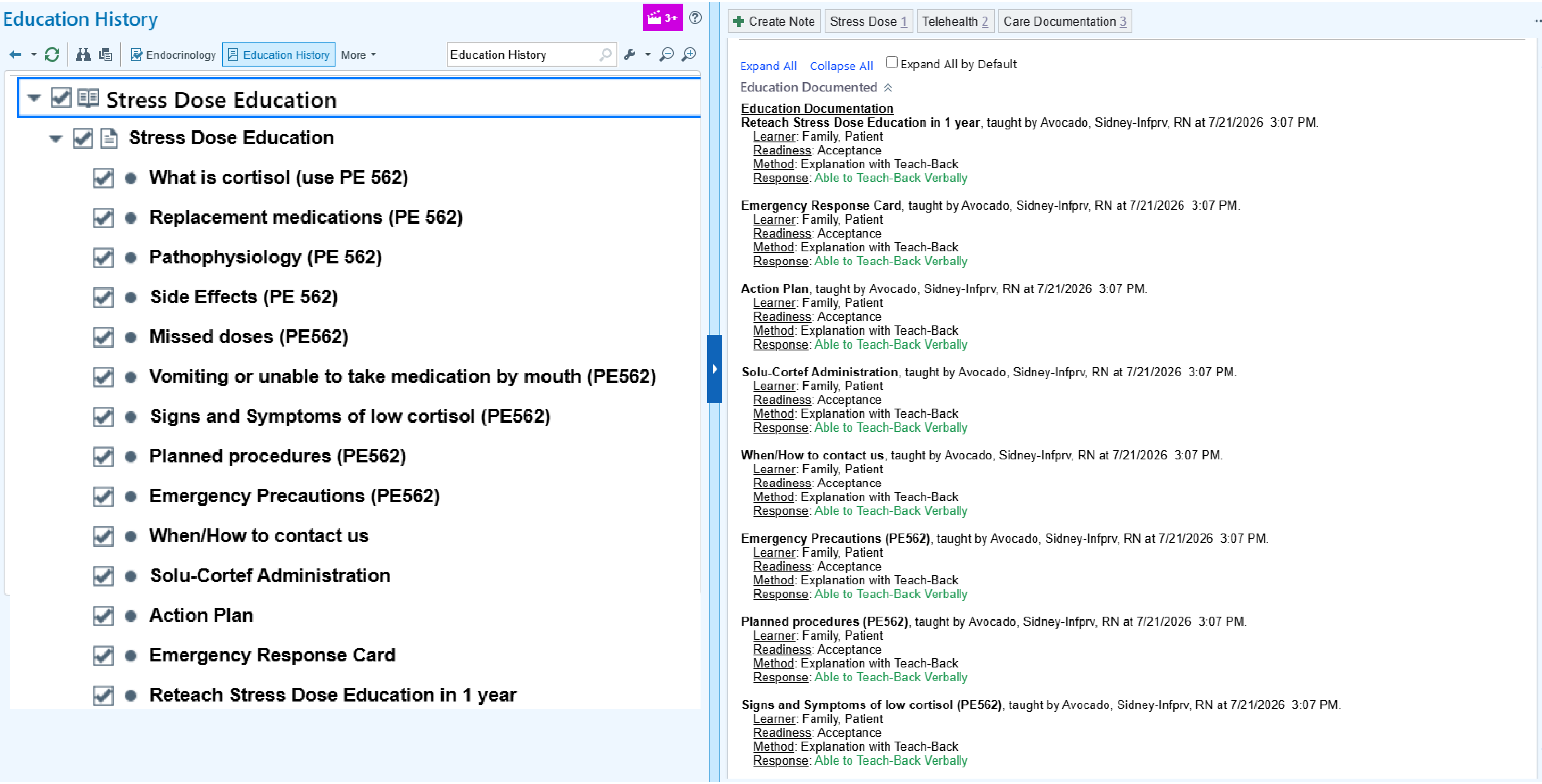

Supplementary figure. Adrenal insufficiency nursing education flowsheet

© 2026 Epic Systems Corporation
